# Correction: Bischoff et al. The Effects of the Food Additive Titanium Dioxide (E171) on Tumor Formation and Gene Expression in the Colon of a Transgenic Mouse Model for Colorectal Cancer. *Nanomaterials* 2022, *12*, 1256

**DOI:** 10.3390/nano13212888

**Published:** 2023-10-31

**Authors:** Nicolaj S. Bischoff, Héloïse Proquin, Marlon J. Jetten, Yannick Schrooders, Marloes C. M. Jonkhout, Jacco J. Briedé, Simone G. van Breda, Danyel G. J. Jennen, Estefany I. Medina-Reyes, Norma L. Delgado-Buenrostro, Yolanda I. Chirino, Henk van Loveren, Theo M. de Kok

**Affiliations:** 1 Department of Toxicogenomics, GROW School for Oncology and Reproduction, Maastricht University Medical Center, 6229 ER Maastricht, The Netherlands; heloise.proquin@rivm.nl (H.P.); marlon.jetten@maastrichtuniversity.nl (M.J.J.); yannickschrooders@xs4all.nl (Y.S.); marloes.jonkhout@kuleuven.be (M.C.M.J.); j.briede@maastrichtuniversity.nl (J.J.B.); s.vanbreda@maastrichtuniversity.nl (S.G.v.B.); danyel.jennen@maastrichtuniversity.nl (D.G.J.J.); henk.van.loveren@gmail.com (H.v.L.); t.dekok@maastrichtuniversity.nl (T.M.d.K.); 2 National Institute for Public Health and Environment (RIVM), Bilthoven, 3721 MA De Bilt, The Netherlands; 3 Faculty of Health, Medicine and Life Science, Maastricht University Medical Center, 6229 ES Maastricht, The Netherlands; 4 Laboratory of Biosignaling & Therapeutics, Department of Cellular and Molecular Medicine, KU Leuven, 3000 Leuven, Belgium; 5 Laboratorio de Carcinogénesis y Toxicología, Unidad de Biomedicina, Facultad de Estudios Superiores Iztacala, Universidad Nacional Autónoma de México, Mexico City 54090, Mexico; medinaingrid0@gmail.com (E.I.M.-R.); irasemachirino@gmail.com (Y.I.C.)

## 1. Affiliation Correction

In the published publication [1], there was an error regarding the affiliation for Yannick Schrooders (yannickschrooders@xs4all.nl). The affiliation 4 was wrongly added, and the updated affiliation only includes affiliation 1. The authors state that the scientific conclusions are unaffected.

## 2. Text Corrections

There was an error in the original publication [1]. The original publication stated that lymphoid hyperplasia was observed in the histopathology samples of the pilot study. The identified areas showed hyperplasia and anaplasia but did not contain lymph nodes, hence no lymphoid hyperplasia was present. Due to the misinterpretation of the histological images, some minor text corrections are necessary. The authors state that the scientific conclusions are unaffected.

### 2.1. A Correction Has Been Made to the Abstract

In the Abstract the following sentence:

“A pilot study showed that E171 exposed mice developed colorectal adenocarcinomas, which were accompanied by enhanced hyperplasia in epithelial cells, lymphatic nodules at the base of the polyps, and increased tumor size.”

has been changed to:

“A pilot study showed that E171 exposed mice developed colorectal adenocarcinomas, which were accompanied by enhanced hyperplasia in epithelial cells, and increased tumor size.”

### 2.2. A Correction Has Been Made to Section 3.2. Pilot Study: Histopatholog

In Section 3.2 the sentence:

“Mice exposed to E171 showed adenocarcinomas with enhanced hyperplasia in epithelial cells as well as lymphatic nodules at the base of the polyps.”

has been changed to:

“Mice exposed to E171 showed adenocarcinomas with enhanced hyperplasia in epithelial cells as well as epithelial cell infiltration in the muscle layer.”

The Correction also includes the insertion of Figure S1 in Supplementary Materials.

Additionally, Figure 2 from the original publication has been replaced with the adapted version. Scale bars have been added to all images and a zoomed-in version of the top-right corner image has been added. The correct Figure 2 caption was also provided. The corrected Figure 2 appears below.

### 2.3. A Correction Has Been Made to Section 4. Discussion

In Section 4 the sentence:

“The pilot study showed histopathological changes in the colon of this Tg mouse model, including hyperplasia around the epithelial cells and the lymphoid nodules.”

has been replaced with the following sentence:

“The pilot study showed histopathological changes in the colon of this Tg mouse model, including hyperplasia around the epithelial cells and the invasion of epithelial cells into the muscle layer.”

The original part of the main text below:

“Histological examination of colonic specimens from the pilot study revealed an increase in tumor size and enhanced hyperplasia at the bottom of the adenocarcinomas, as well as in lymph nodes. Lymphoid hyperplasia in humans is a rare and benign condition, that can be distinguished in follicular hyperplasia which resembles a stimulation of the B cell compartment, and paracortical hyperplasia, which stimulates the T cell compartment of the affected lymph nodes [31,32]. The pathogenesis of nodular lymphoid hyperplasia is largely unknown, but it is associated with immunological responses such as T cell invasion and immunodeficiency processes [33]. In humans, the presence of nodular hyperplasia is suspected to be a marker for low-grade inflammation in irritable bowel syndromes and generally is associated with gastrointestinal immune responses, such as immunoglobulin G deficiency syndrome and food sensitivities [34]. We hypothesize that the presence of nodular lymphoid hyperplasia in this Tg mouse model could be an indicator of low-grade inflammation and contribute to the increased development of tumors and their size. Recent publications postulated an activation of the NLRP3 inflammasome following the ingestion of E171 [7,35]. The stimulation of the NLRP3 inflammasome results in the release of interleukine-1ß (IL-1ß) and IL-18 and potentially links the observed increases in histopathological changes in the colonic tissues to the induction of inflammatory and immunological responses after E171 exposure [36,37]. Similarities can be found in another Tg mouse model overexpressing IL-1ß, which showed a prominent development of nodular hyperplasia and changes to the microarchitecture of the lymphoid tissues, accompanied by T cell invasion [38].

The tumor formation study indicates an earlier onset of tumor formation and differences in tumor size and number, following the exposure to E171. The occurrence of these events might be explained by the findings of our gene expression study.”

has been replaced with the following:

“Histological examination of colonic specimens from the pilot study revealed an increase in tumor size and enhanced hyperplasia at the bottom of the adenocarcinomas. We observed large hyperplastic areas, showing loss of tissue architecture, nuclear enlargement, and increased nucleus-to-cytoplasm ratio (anaplasia), but also infiltrated epithelial cells in the muscle layer, which denotes malignancy. Although infiltrated epithelial cells were observed in both, control and treated mice, the amount of cell infiltration was higher in E171 exposed mice [31].

The tumor formation study indicates an earlier onset of tumor formation and differences in tumor size, following the exposure to E171. The occurrence of these changes in tissue architectures and tumors size might be explained by the findings of our gene expression study.”

### 2.4. A Correction Has Been Made to Section 5. Conclusions

The Section 5 the sentence:

“In this comprehensive study, we found a statistically nonsignificant increase in tumor formation and progression induced by E171 in a *CAC^Tg/Tg^;APC^580S/+^* transgenic mouse model for colorectal cancer.”

has been corrected as follows:

“In this comprehensive study, using a *CAC^Tg/Tg^;APC^580S/+^* transgenic mouse model for colorectal cancer, we showed increased tumor growth and progression, but found no statistically significant increase in tumor formation induced by E171.”

### 2.5. A Correction Has Been Made to the References

With the corrections described above, the reference citations in the main text as well as the order of some references have been adjusted accordingly. The references [31–38] from the original work have been removed and a new reference [31] has been added. New reference [31]: Lim, C.S.; Kim, E.S.; Kim, J.Y.; Hong, S.T.; Chun, H.J.; Kang, D.E.; Cho, B.R. Measurement of the Nucleus Area and Nucleus/Cytoplasm and Mitochondria/Nucleus Ratios in Human Colon Tissues by Dual-Colour Two-Photon Microscopy Imaging. *Sci. Rep.* **2015**, *5,* 18521. https://doi.org/10.1038/srep18521.

The authors state that the scientific conclusions are unaffected. This correction was approved by the Academic Editor. The original publication has been updated.

## Figures and Tables

**Figure 2:**
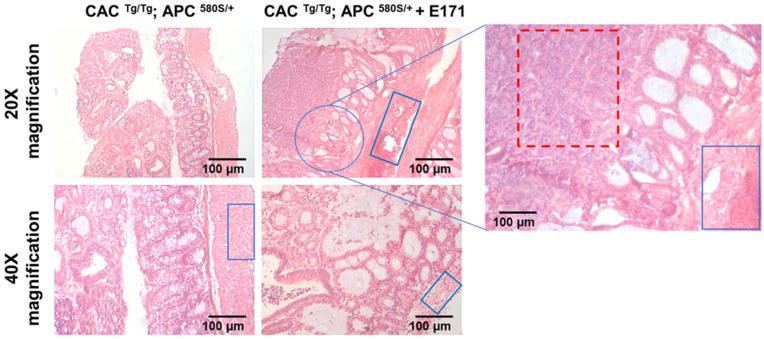
Histopathological analysis of tumors in control and E171-treated mice showed well-differentiated adenocarcinomas. The mice exposed to E171 additionally showed enhanced hyperplasia in the epithelial cells as well as epithelial cell infiltration in the muscle layer of the adenocarcinomas. Red squares—epithelial cell hyperplasia and anaplasia; blue squares—epithelial cell infiltration into the muscle layer (hyperplasia).
